# SEOM Clinical Guideline of management of soft-tissue sarcoma (2016)

**DOI:** 10.1007/s12094-016-1574-1

**Published:** 2016-11-29

**Authors:** A. López-Pousa, J. Martin Broto, J. Martinez Trufero, I. Sevilla, C. Valverde, R. Alvarez, J. A. Carrasco Alvarez, J. Cruz Jurado, N. Hindi, X. Garcia del Muro

**Affiliations:** 1 Servicio de Oncología Médica, Hospital de la Santa Creu i Sant Pau, Mas Casanovas 90, 08041 Barcelona, Spain; 2 Hospital Virgen del Rocio, Seville, Spain; 3 Hospital Universitario Miguel Servet, Saragossa, Spain; 4 Hospital Universitario Virgen de la Victoria, Malaga, Spain; 5 Hospital Vall d’Hebro, Barcelona, Spain; 6 Hospital Universitario Gregorio Marañon, Madrid, Spain; 7 Hospital Universitario de Vigo, Vigo, Spain; 8 Hospital Universitario de Canarias, Tenerife, Spain; 9 Hospital Duran I Reynals, Hospitalet de Llobregat, Spain

**Keywords:** Sarcoma, Soft-tissue tumors, Clinical guidelines, Uncommon tumors

## Abstract

Soft-tissue sarcomas are uncommon and heterogeneous tumors of mesenchymal origin. A soft-tissue mass that is increasing in size, greater than 5 cm, or located under deep fascia are criteria for suspicion of sarcoma. Diagnosis, treatment, and management should preferably be performed by a multidisciplinary team in reference centers. MRI and lung CT scan are mandatory for local and distant assessment. A biopsy indicating histological type and grade is needed previous to the treatment. Wide surgical resection with tumor-free tissue margin is the primary treatment for localized disease. Radiotherapy is indicated in large, deep, high-grade tumors, or after marginal resection not likely of being improved with reexcision. Neoadjuvant and adjuvant chemotherapy improve survival in selected cases, usually in high-grade sarcomas of the extremities. In the case of metastatic disease, patients with exclusive lung metastasis could be considered for surgery. First-line treatment with anthracyclines (or in combination with ifosfamide) is the treatment of choice. New drugs have shown activity in second-line therapy and in specific histological subtypes.

## Introduction

Soft-tissue sarcomas (STS) constitute an uncommon and heterogeneous group of tumors of mesenchymal origin, with an estimated incidence of five cases per 100,000 people per year in Europe. Although STS comprise different histopathological subtypes (more than 50 according the 2013 WHO classification), they share several clinical and pathological features and are usually considered as a group for diagnostic and therapeutic purposes, with the exception of specific particularities of some subtypes, such as rhabdomyosarcoma, gastrointestinal stromal tumors, extraosseus osteosarcoma, and Ewing’s sarcoma. STS can arise anywhere in the body, but most originate in the extremities, less frequently in the trunk, retroperitoneum, head and neck, and viscera. They can occur at any age, and although more common in middle aged and older adults, they are also seen in children and young adults.

## Methodology

These guidelines have been developed by a group of medical oncologists with expertise in sarcoma research, diagnosis, and therapy. A bibliographic search of published articles was performed in the MEDLINE database (PubMed). Searches were limited to human studies, clinical trials, meta-analyses, clinical guidelines, and consensus statements. In addition, a review of abstracts of relevant, which do not published, yet phase III studies focused on STS therapy presented at international oncology meetings as the American Society of Clinical Oncology (ASCO), European Society of Medical Oncology (ESMO) and Connective Tissue Oncology Society (CTOS) meetings, in the recent years, was performed. First, the different sections were written by different responsible experts and after all the members discussed the results and determined the level of evidence and the grade for each recommendation according to ESMO guidelines. The main objective of this document consists of providing clear practical recommendations about the different aspects involved in the management of this group of diseases, intended to help in the therapeutic decision-making processes, and, therefore, contributes to improve STS patient’s care in Spain.

## Diagnosis and staging

### Warning signs and referral recommendations

Criteria for suspicion of STS and the need to contact with a reference center, is a soft tissue mass often painful, greater than 5 cm or progressively increasing in size, located under the deep fascia or that relapse after an inadvertent excision. STS require a multidisciplinary therapeutic approach, involving pathologists, radiologists, surgeons, radiation therapists, and medical oncologists. The early recognition and referral to a specialist center that provides a multidisciplinary diagnosis and therapeutic approach, treating a high number of cases annually, improves the outcome of the patients with STS [1]. Preferably, biopsy should be done at the same center of treatment, and the resection of the biopsy trajectory must be performed in the definitive surgery.

### Imaging studies, local and distant staging

Magnetic resonance imaging (MRI) is the method of choice for the initial study for tumors arising in limbs, trunk wall, and pelvis. MRI should be performed prior to biopsy, to avoid changes due to biopsy and should provide information about size, location (depth, compartments), lesion limits, perilesional edema, and relation to neurovascular structures, and suggest the biopsy area. Contrast-enhanced multi-slice computed tomography (CT) is the best choice in intraabdominal or retroperitoneal STS, in the case of MRI contraindication or to assess bone involvement. A chest CT scan is mandatory to exclude pulmonary metastases.

TNM system is the most frequently used staging system for soft-tissue sarcomas; it includes tumor size, depth (superficial or deep), lymph node involvement, presence of distant metastases, and histological grade to determine stage.

### Planned biopsy, histological, and molecular diagnosis

Core-needle biopsy guided by imaging (ultrasound or CT scan) is the preferred method of biopsy. It is mandatory to avoid non-involved anatomical compartments, and it should be kept in mind that the path of the biopsy must be resected by definitive surgery. Incisional biopsy is an alternative in the cases, where needle biopsy is not feasible. Excisional biopsy is only acceptable for superficial lesions smaller than 3 cm in size. Cytology could be useful in detecting recurrences, but it should not be used in the diagnosis of STS [2].

A histological diagnosis should be made according to the 2013 WHO Classification of STS. The histological grade following the FNCLCC-grading system should be provided, with the exception of some specific sarcoma types, where the aggressiveness is defined by the histological type itself. The pathological report of the surgical sample should include the following items: surgical procedure, location, size, histological type, histological grade, margins, invasion of adjacent structures, immunohistochemistry and molecular techniques performed, and percentage of necrosis if preoperative treatment is administered. In addition, molecular diagnosis by detection of translocations and their fusion genes by RT-PCR or FISH could be useful in uncertain diagnosis, uncommon presentations and variants, as well as in the cases, where the results may have a prognostic or predictive relevance or implications in the treatment [3].

## Treatment of localized disease

### Surgery for soft-tissue sarcomas

Mainstay of therapy for localized soft-tissue sarcoma is surgical resection. Biopsy should always be performed before surgery by a specialized team preferably with radiologic guidance. “*En bloc*” wide resection of the lesion with negative resection margins (II, A) should be performed by an experienced surgeon (III, A) based on the decision of a multidisciplinary board. Wide resection can sometimes be facilitated by reconstructive surgeries. The size of the adequate margin depends on several factors (size of the tumor, neoadjuvant treatment, location, grade, and adjacent structures). At least, a 1 cm margin or an intact anatomic barrier (periostium, epineura, vascular, or muscular fasciae) is recommended. Several kinds of surgery can be performed:

1. Amputation and/or disarticulation should be reserved only when complete resection with conservative surgery is not feasible, specially in the case of wide infiltration of the neurovascular bundle, or the risk of nonfunctional member.
2. Wide resection that includes the tumor and an appropriate margin, or compartmental resection (that could be associated with functional defects) are the recommended procedures.
3. Marginal excision includes peritumoral reactive tissue but not enough margins and is associated with high local recurrence rates. Only acceptable in some cases of atypical lipomatous tumors (IV, B).

In the cases of positive margins after surgery, if wide margins can be obtained without major morbidity, re-excision is recommended. The use of radiotherapy does not compensate for positive margins.

Surgical incision should follow the longitudinal axis of the member and the previous biopsy tract should be included. Diagnostic lymph node dissection is not a standard practice. In cases, where regional lymph nodes are positive, surgery should also include their resection (lymphadenectomy) (III, B). If an exclusive local recurrence occurs, salvage surgery should be attempted [4].

### Radiotherapy treatment in Localized disease in STS

Complementary radiotherapy (RT) can be offered in addition to surgery to optimize local control. Two prospective randomized trials, one using brachytherapy (BRT) and the other one with postoperative external beam RT (EBRT), demonstrated the local control advantage of adjuvant RT over surgery alone in sarcomas. Their results showed a statistically significant reduction in local recurrences, without significant differences in the overall survival. Based on these studies, adjuvant radiotherapy is recommended following wide resection in high grade (G2–3), deep, and larger than 5 cm sarcomas, as well as in those either resected with close margins, or locally recurrent high grade without prior radiation. Radiotherapy could be omitted in most patients with low-grade sarcoma, in small or superficial tumors with wide resection margins, and when compartmental surgery or amputation has been performed. In the remaining situations, the administration of radiotherapy should be assessed individually regarding the local recurrence risk.

Radiotherapy is most commonly administered postoperatively, at doses of 60–66 Gy. However, preoperative radiotherapy at doses of 50 Gy constitutes an acceptable alternative, since a phase III study showed similar efficacy to postoperative radiotherapy, with less long-term fibrosis and edema but increased wound complications. In the adjuvant setting, both EBRT and BRT techniques have shown to be equally useful. Recently, some data have suggested that new technics of RT could improve therapeutic ratio: Intensity-modulated RT (IMRT) has been compared in a nonrandomized way at a single institution showing less toxicity and better local control than EBRT, and image-guided RT was evaluated in RTOG-0630 trial in 98 patients treated preoperatively showing better toxicity profile than historical data with EBRT [5, 6] (III, B).

### Adjuvant and neoadjuvant chemotherapy

Adjuvant chemotherapy remains controversial, since the results of different randomized trials are non-conclusive. An updated meta-analysis, however, showed a significant but limited benefit in survival. This effect was more pronounced in the subgroup of patients who received anthracyclines and ifosfamide [7]. For this reason, it constitutes a standard option of treatment in selected patients (II, A). Its administration should be only considered in those patients with high grade, deep and >5 cm tumors, especially if they are located in the extremities (II, A). If chemotherapy is administered, a regimen, including anthracyclines and ifosfamide, is recommended (II, A). Although the recommendation consists of five cycles, the results of a randomized trial of neoadjuvant chemotherapy showed non inferiority of three cycles compared to five. Long-term results confirmed that these results in a perioperative setting [8] (II, B).

The number of studies regarding neoadjuvant chemotherapy in STS is limited, and most of them are small series and phase II trials. A randomized non-inferiority phase III study compared three cycles of pre-operative chemotherapy with epirubicin and ifosfamide versus the same regimen plus two additional adjuvant cycles of treatment after surgery. Long-term results showed that three cycles were not inferior to five cycles in terms of recurrence and survival [8]. A recently reported phase III trial of neoadjuvant chemotherapy fails to show an advantage of histology-tailored chemotherapy over the standard chemotherapy with epirubicin and ifosfamide in resectable high-risk STS of the extremities or trunk wall. However, the presence of a statistically significant and clinically relevant difference in RFS and OS at >3 years in favour of the standard chemotherapy provides strong, randomized evidence in support of neoadjuvant chemotherapy [9] (I, B).

Therefore, despite the current setting of shortage of evidence, some practical recommendations regarding neoadjuvant therapy in STS may be made. It could be considered as an option in those cases with high grade, deep, and large (>5 cm) STS that are marginally resectable or require very aggressive surgery without assuring clean margins (III, B). In those cases, probably, the combination of pre-operative radiation and chemotherapy might have advantages over either modality alone (IV, B).

### Role of isolated limb perfusion and hyperthermia

**Fig. 1:**
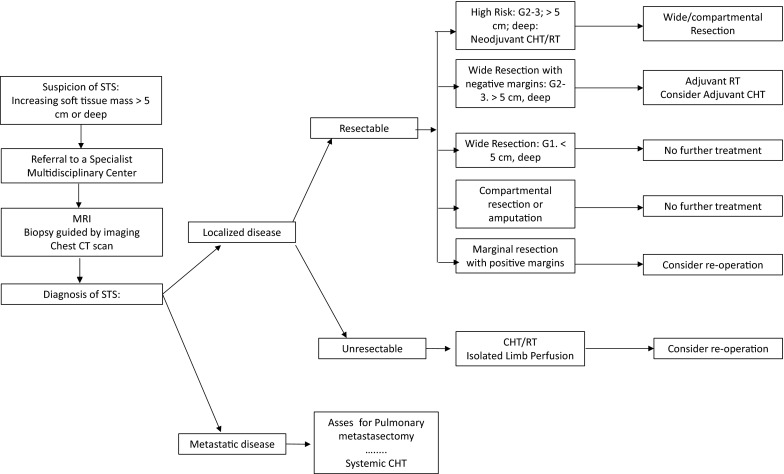
Primary management of STS algorithm

Hyperthermic-isolated limb perfusion (ILP) with tumor necrosis-factor alpha and melphalan (III, B) or regional hyperthermia combined with chemotherapy [10] (I, B) may be considered in patients with limb STS when conservative surgery is not feasible for locally advanced disease or in palliative setting, to avoid mutilating surgery (Fig. 1).

## Treatment of advanced disease

With the current appropriate management, local control is achieved in around 80–90% of patients. However, approximately half of patients with high-grade tumors will develop metastatic disease and could die from the disease.

### Surgery

Patients with exclusive pulmonary metastasis should be evaluated for surgery. The decision should be based on the disease-free period following primary surgery (ideally greater than 1 year) and the possibility of resection with negative margins rather than the number of lesions (III, B). Complete resection of pulmonary metastases in these selected patients achieves up to 20% long-term survival [11]. Prior re-staging should be performed to rule out other sites of disease. There is no clear evidence of the benefit of “adjuvant” chemotherapy after resection of metastases in STS. In contrast, in patients with synchronous lung metastases, short disease-free interval, or high number of lesions, chemotherapy should be the initial treatment. Subsequent surgery could be an option if benefit is achieved from chemotherapy (IV, C).

### Radiotherapy

Beyond the indication of radiotherapy as a palliative treatment for local or metastatic sites to control pain or other symptoms, the use of stereotactic body radiation therapy (SBRT) for lung metastases has shown excellent local control rates (above 80% at 5 years) with limited toxicities [12]. Thus, SBRT should be evaluated in a multidisciplinary team for patients unfit for surgery (III, B).

### Systemic treatment

#### First line

- *Doxorubicin* and *ifosfamide* are the most active drugs and constitute the standard treatment for advanced STS. The association of doxorubicin and ifosfamide increased the response rate and toxicity but did not significantly improve survival in randomized trials [13] (I, A). Therefore, the recommended first-line treatment is doxorubicin at 75 mg/m^2^. Ifosfamide at 6–12 g/m^2^ could be an alternative in case of doxorubicin contraindication, or as a second-line treatment after doxorubicin failure. However, the use of a combination regimen of both drugs could be justified when obtaining an objective response to improve symptoms or resectability is important (II, B).
- *Olaratumab* in combination with Doxorubicin is the most recently approved drug by the FDA and EMA in STS patients. A randomized phase II trial that include anthracycline-naive patients, although 55% of them had received chemotherapy, showed benefit in PFS (6.6 months with olaratumab plus doxorubicin and 4.1 months with doxorubicin HR 0.67, *p* = 0.0615), and median OS (26.5 months with olaratumab plus doxorubicin and 14.7 months with doxorubicin (HR 0.46, *p* = 0.0003) (II, B) [14].

#### Second-line chemotherapy and beyond

Second-line therapy for advanced or metastatic unresectable disease is always palliative. Thus, close clinical observation may be an option for asymptomatic patients, especially for those with low-grade tumors or known low responsive entities (IV, D). Symptomatic patients with good performance status are good candidates for clinical trials. If not available, the conventional systemic therapy should be offered:

- *Trabectedin* has shown a modest objective response rate but a higher progression arrest rate, especially in liposarcoma (LPS) (notably myxoid LPS, PFS at 6 months of 88%) and leiomyosarcoma (LMS), but also in other tumor types. It was approved in Europe for patients with sarcoma after progression to doxorubicin and ifosfamide or in patients ineligible for these treatments, and more recently in USA after a phase III trial showed that trabectedin improved disease control in comparison with DTIC (median PFS 4.2 *v* 1.5 months), in advanced pre-treated metastatic LPS or LMS [15] (I, A). It should be administered at 1.5 mg/m^2^ over 24 h every 21 days with dexametasone and through a central venous access.
- *Pazopanib* constitutes an appropriate option in non-adipocitic sarcoma (I, A) based on the positive results in terms of median PFS and disease stabilization (4.6 versus 1.6 months, 67 versus 38%, respectively) of a phase III study (PALETTE trial) [16] comparing pazopanib (800 mg daily) versus placebo in patients with non-adipocitic sarcomas progressing after first-line chemotherapy. All the included subtypes seemed to benefit to the same extent. Given the risk for serious hepatotoxicity, close monitoring of liver function tests is recommended, particularly in the first 9 weeks of therapy.
- *Eribulin* was approved in Europe for LPS patients, after or intolerant to anthracycline containing therapy, based on the benefit observed in a phase III randomized trial in terms of the overall survival over DTIC (13.5 versus 11.5 months for the total population and 11.6 versus 8.4 months for LPS), although no significant differences were seen in PFS or RR [17] (I, A). It should be administered at 1.4 mg/m^2^ in 2–5 min, days 1 and 8 every 21 days.
- *Gemcitabine* and *DTIC* have been evaluated in monotherapy in several phase II trials showing limited activity (II, B). However, the superiority of the combination of gemcitabine (1800 mg/m^2^ at 10 mg/m^2^/min) with DTIC (500 mg/m^2^) every 14 days versus DTIC alone has been reported in a randomized phase II trial in terms of median PFS and overall survival, especially in LMS [18] (II, B).
- *Docetaxel* in combination with gemcitabine has demonstrated interesting responses, especially in uterine LMS in randomized phase II trials versus gemcitabine alone (RR 16 versus 8% and superior median PFS and median OS) [19]. Patients with LMS and undifferentiated pleomorphic sarcoma appeared to get the greatest benefit (II, C).
- As a dose–response relationship has been shown for *ifosfamide*, patients who have previously received ifosfamide may be rescued with *high*-*dose ifosfamide* (>10 g/m^2^) [20] (III, B). Particular sensitivity has been reported for synovial sarcoma.

For the majority of STS, there is no evidence that a particular drug sequence is better than another and probably most patients with good performance status benefit from being exposed to the largest number of available drugs (IV, B).

## Therapeutic considerations for specific STS subtypes

### Retroperitoneal sarcomas

Retroperitoneal sarcomas are characterized by poor prognosis. More than half are high-grade and adequate surgical margins are rarely obtained. The standard imaging procedure is a chest-abdominal CT scan (V, A). An extraperitoneal image-guided percutaneous core needle biopsy is used for histologic diagnosis (IV, A). Nevertheless, it is reasonable to avoid biopsy if the imaging is pathognomonic (heterogeneous dedifferentiated/well-differentiated LPS) and no preoperative treatment is planned (V, A) *En bloc* resection of the tumor, including adherent structures even if not overtly [21] infiltrated at the time of primary presentation, is the only curative treatment for RPS (III, A). Post-operative radiation therapy is of limited value and not a standard treatment, could be associated with great toxicity, and may be an option in highly selected patients with well-defined risk areas of recurrence (IV, C). Adjuvant and neoadjuvant chemotherapy should not be routinely employed in RPS due to lack of evidence of benefit (IV, C).

### Uterine sarcomas

Uterine sarcomas are composed of different tumor entities: leiomyosarcomas, high-grade uterine sarcoma, and endometrial stromal sarcoma (ESS). Carcinosarcomas behave like epithelial carcinomas and are not covered by the following guidelines. Standard surgery of localized US consists of total abdominal hysterectomy (plus double oophorectomy only in ESS) with full abdominal cavity exploration. Lymphadenectomy is not indicated. Adjuvant radiotherapy is controversial. Most available data are retrospective and suggest an improvement in local relapse control but not consistent improvement in overall survival [22]. Thus, adjuvant radiotherapy it is not routinely considered, but it could be recommended in selected cases with a high relapse risk (II, C). There is not enough evidence to support the use of adjuvant chemotherapy, but it could be individually planned in some patients with high risk of systemic relapse (III, B). Hormonal therapy with megestrol acetate, gonadotropin-releasing hormone (GnRH) analogues, and aromatase inhibitors can delay progression for long periods of time in low-grade oestrogen receptor-positive ESS, and it is preferred over chemotherapy as front-line palliative treatment (IV, C). Doxorubicin is an active single agent for US and is less toxic than combination regimens; for that reason, it constitutes the standard first-line treatment for advanced US (I, A). Positive results have been published for LMS patients treated with gemcitabine plus docetaxel as first- or second-line treatment. It is acceptable to select this regimen as first-line palliative chemotherapy (III, B). Systemic treatment in second and further line is similar to other STS.

### Desmoid tumors

Desmoid tumors represent a mesenchymal neoplasm of intermediate behavior. They do not metastasize, but show a marked tendency to local relapse. Surgery has classically been the mainstay of DT curative treatment. It is usually straight forward in the case of limb and chest-wall tumors, but can be much more challenging in abdominal disease. The aim of surgery is the macroscopic removal of the whole tumor while minimizing morbidity [23]. Wide margins, even microscopically negative ones, do not justify on their own mutilating surgeries or functional sequels, as the prognosis of macroscopically resected (R1) patients do not depend on the microscopical status of the margins (III, A). Given the unpredictable natural history of the disease and functional problems implied by some tumor locations, a watch-and-wait approach is also acceptable (III, B). For progressing cases, the optimal treatment needs to be individualized. RT is able to control even bulky disease for long periods of time. (III, B). Systemic treatment is appropriate for unresectable tumors, Gardner-related cases with multiple recurring DT, progressions in areas previously irradiated, and functionally or aesthetically unacceptable surgery. Evidence-based options include non-steroidal anti-inflammatory drugs, such as sulindac (IV, D), anti-oestrogens (tamoxifen and toremifene) (IV, D), chemotherapy (low-dose methotrexate plus vinblastine or vinorelbine, liposomal doxorubicin and vinorelbine monotherapy) (III, B), imatinib (III, B), sorafenib (III, B), and full-dose chemotherapy (III, C). We recommend to employ the less toxic monotherapy options in the first place.

### Dermatofibrosarcoma protuberans

Dermatofibrosarcoma protuberans is a cutaneous mesenchymal tumor of intermediate behavior that rarely metastasizes but is locally aggressive. The treatment of localized DFSP is wide surgical excision with wide margins (2–4 cm). Mohs surgery can be planned to avoid major cosmetic defects (III, B). Adjuvant radiation therapy should be considered when margins are positive and re-resection is not feasible (IV, B). In unresectable, recurrent or metastatic DFSP, imatinib is recommended [24] (III, B). Imatinib activity is related to the presence of *t*(17;22) translocation. However, objective responses have been documented in translocation-negative tumors. If transformation to high-grade sarcoma occurs (less than 15% of DFSP), management is similar than a conventional high-grade STS.

### Other rare specific subtypes

In metastatic or locally advanced malignant, **solitary fibrous tumor** antiangiogenic agents, such as sunitinib (III, B) or the combination of temozolomide plus bevacizumab, constitute active options [25] (IV, B). Chemotherapy following the common guidelines for STS could be administered but its efficacy is low (III, B). **Alveolar soft part sarcoma** is not particularly sensitive to classic chemotherapeutic agents. However, ASPS has an upregulation of angiogenesis elements, and cediranib has proven to be active in advanced disease (III, B). Several partial responses to sunitinib and bevacizumab have also been reported (IV, B). **Clear cell sarcoma** tends to metastasize to lymph nodes, unlike other STS. In advanced cases, the efficacy of chemotherapy is low. However, some isolated responses have been described with antiangiogenic agents, such as sorafenib or sunitinib (V, D). The **PEComa** family of tumors consists of related mesenchymal neoplasms that share a distinctive cell type, the perivascular epithelioid cell. It includes angiomyolipoma, clear cell “sugar” tumor of the lung, lymphangioleiomyomatosis, clear cell myomelanocytic tumor of the falciform ligament/ligamentum teres, abdominopelvic sarcoma of perivascular epithelioide cells, and extrapulmonary sugar tumor. Although most PEComas are benign, a subset exhibits malignant behavior. Frequently, tumors of the PEComa family share dysregulated activation of the mechanistic target of rapamycin (mTOR) signaling through mutations in the *TSC1* or *TSC2* genes. This is the basis for the use of mTOR inhibitors, sirolimus, or everolimus in the treatment of locally invasive or metastatic PEComas (IV B). The** Inflammatory myofibroblastic tumor** (IMT) is associated with rearrangements of the ALK (anaplastic lymphoma kinase) locus on chromosome 2p23.13. In advanced IMT ALK-translocated, ALK-inhibitors, as crizotinib, produce sustained responses and constitute the best option (IV, B). Finally,** Angiosarcoma** (AS) is a heterogeneous type of sarcoma due to its age of presentation and location. In advanced cases, systemic chemotherapy with either anthracyclines or taxanes is acceptable treatment options (II B). However, in the AS of the scalp, frequently seen in elderly patients, weekly paclitaxel is the drug of choice, because it seems to have a better response rate than anthracyclines. Antiangiogenic drugs, such as bevacizumab and sorafenib, have also been tested in metastatic AS with moderate activity [26] (III, B).
